# Comparative Secretome and Functional Analyses Reveal Glycoside Hydrolase Family 30 and Cysteine Peptidase as Virulence Determinants in the Pinewood Nematode *Bursaphelenchus xylophilus*

**DOI:** 10.3389/fpls.2021.640459

**Published:** 2021-03-08

**Authors:** Ryoji Shinya, Haru Kirino, Hironobu Morisaka, Yuko Takeuchi-Kaneko, Kazuyoshi Futai, Mitsuyoshi Ueda

**Affiliations:** ^1^School of Agriculture, Meiji University, Kawasaki, Japan; ^2^Graduate School of Agriculture, Kyoto University, Kyoto, Japan

**Keywords:** pinewood nematode, secreted protein, virulence determinant, *Nicotiana benthamiana*, glycoside hydrolase family 30, cysteine peptidase

## Abstract

Pine wilt disease, caused by the pinewood nematode, *Bursaphelenchus xylophilus*, is one of the world’s most serious tree diseases. Although the *B*. *xylophilus* whole-genome sequence and comprehensive secretome profile have been determined over the past decade, it remains unclear what molecules are critical in pine wilt disease and govern *B. xylophilus* virulence in host pine trees. Here, a comparative secretome analysis among four isolates of *B. xylophilus* with distinct virulence levels was performed to identify virulence determinants. The four candidate virulence determinants of *B. xylophilus* highly secreted in virulent isolates included lipase (Bx-lip1), glycoside hydrolase family 30 (Bx-GH30), and two C1A family cysteine peptidases (Bx-CAT1 and Bx-CAT2). To validate the quantitative differences in the four potential virulence determinants among virulence groups at the protein level, we used real-time reverse-transcription polymerase chain reaction analysis to investigate these determinants at the transcript level at three time points: pre-inoculation, 3 days after inoculation (dai), and 7 dai into pine seedlings. The transcript levels of *Bx-CAT1*, *Bx-CAT2*, and *Bx-GH30* were significantly higher in virulent isolates than in avirulent isolates at pre-inoculation and 3 dai. A subsequent leaf-disk assay based on transient overexpression in *Nicotiana benthamiana* revealed that the GH30 candidate virulent factor caused cell death in the plant. Furthermore, we demonstrated that Bx-CAT2 was involved in nutrient uptake for fungal feeding via soaking-mediated RNA interference. These findings indicate that the secreted proteins Bx-GH30 and Bx-CAT2 contribute to *B. xylophilus* virulence in host pine trees and may be involved in pine wilt disease.

## Introduction

Pine wilt disease, caused by the pinewood nematode (PWN), *Bursaphelenchus xylophilus*, is one of the most serious tree diseases worldwide because pine forests are essential sources of forest products. Moreover, pine trees are used in wind-, tide-, and sand-breaks, as well as in landslide prevention and to beautify landscapes. However, pine forests face a catastrophic threat from pine wilt disease in East Asian countries. The disease was first discovered in the early 20th century in Japan, following its introduction from the United States, and *B*. *xylophilus* was identified as the etiologic agent of the disease in 1971 (Kiyohara and Tokushige, 1971). In the 1980s, the pathogen spread to neighboring East Asian countries (Yi et al., 1989), and was subsequently isolated in Portugal in 1999 (Mota et al., 1999) and in Spain in 2008 (Abelleira et al., 2011). Because of active international trade in forest products, the PWN poses a potentially serious problem in both the Southern and Northern Hemispheres. Thus, the PWN is currently a worldwide threat.

There is a significant variation among *B. xylophilus* isolates in terms of level of virulence in pine trees (Kiyohara and Bolla, 1990; Akiba, 2006). Virulent isolates of *B. xylophilus* can effectively kill pine seedlings when thousands of nematodes are inoculated into 2–3-year-old pine seedlings. However, avirulent isolates typically kill pine seedlings only when host pines are exposed to stresses such as low light conditions (Ikeda, 1996). In addition to differences in virulence to host pines, remarkable differences have been observed in various biological characteristics between virulent and avirulent isolates (e.g., transmission to vector beetles, reproduction in healthy pine trees and on a fungal mat, and tolerance to oxidative stress) (Kiyohara and Bolla, 1990; Aikawa et al., 2003; Aikawa and Kikuchi, 2007; Shinya et al., 2012; Vicente et al., 2015). Several reports investigated the genetic diversity and the difference in gene expression patterns to identify the factors that control these phenotypic differences between virulent and avirulent isolates (Palomares-Rius et al., 2015; Ding et al., 2016; Filipiak et al., 2020). These studies reported candidate molecules that may be related to the virulence of *B. xylophilus* and significant differences in peptidases and antioxidant enzymes between virulent and avirulent isolates. However, the mechanisms that determine the differences in these *B. xylophilus* traits remain unclear due to the lack of effective functional analysis.

Here, we focused on *B. xylophilus* secreted proteins to investigate the mechanisms underlying the differences in these *B. xylophilus* traits, especially virulence in host pines. Secreted proteins of plant-parasitic nematodes are considered important for their parasitism. Several molecules have been suggested as *B. xylophilus* pathogenic factors: cell-wall-degrading enzymes secreted by the PWN (Odani et al., 1985; Kikuchi et al., 2004, 2006), venom allergen-like proteins (Kang et al., 2012), sphingolipid activator protein (Hu et al., 2019), thaumatin-like proteins (Kirino et al., 2020), cysteine protease inhibitors (Kirino et al., 2020), and PWN-associated bacterial toxins (Han et al., 2003; Zhao and Lin, 2005). The previous proteomic and transcriptomic analyses revealed the comprehensive profile and unique characteristics of *B. xylophilus* secreted proteins (Shinya et al., 2013; Cardoso et al., 2016; Espada et al., 2016, 2018). However, the contribution of each molecule to *B. xylophilus* virulence in pines and the development of pine wilt disease remains unclear because no systematic screening of virulence factors has been performed. Moreover, there are few functional analysis tools available for *B. xylophilus* (e.g., transgenesis and CRISPR/Cas9).

In this study, a comparative secretome analysis among four isolates of *B. xylophilus* with distinct virulence levels was performed to identify virulence determinants. We presumed that different expression levels of secreted proteins among groups with disparate virulence would reflect involvement in PWN pathogenicity. Here, we demonstrated that four proteins were highly expressed in virulent isolates. A subsequent leaf-disc assay based on transient overexpression in *Nicotiana benthamiana* revealed that one of four candidate virulent factors, glycoside hydrolase family 30 (GH30), was able to cause plant cell death. Furthermore, we demonstrated that Bx-CAT2 was involved in nutrient uptake for fungal feeding via soaking-mediated RNA interference.

## Materials and Methods

### Nematodes Used in Comparative Proteome Analysis

Four isolates of *B. xylophilus* were used in this study: two virulent isolates (Ka4 and S10) and two avirulent isolates (OKD-1 and C14-5) (Aikawa and Kikuchi, 2007; Zhou et al., 2007). Ka4 and S10 were originally isolated from *Pinus densiflora* in 1994 and 1982, respectively. C14-5 was originally isolated from the Japanese pine sawyer, *Monochamus alternatus*, emerged from *P. densiflora* in 1975 with low virulence (Kiyohara, 1989). OKD-1 was originally isolated from *Pinus thunbergii* in 1984, and its virulence level was decreased during subculturing on the fungus *Botrytis cinerea* (Kawazu et al., 1996). A mixed culture of the propagative forms, including second- (J2), third- (J3), and fourth-stage juveniles (J4), as well as adults and eggs, were propagated on the fungus *B. cinerea* on potato dextrose agar medium (*Nissui*-seiyaku) containing 100 units/ml penicillin and 100 μg/ml streptomycin at 25°C. After incubation for 10 days, the PWNs were extracted from the culture for 6 h using the Baermann funnel technique (Baermann, 1917), then washed 10 times in sterile water containing 100 units/ml penicillin, 100 μg/ml streptomycin, and 0.25 μg/ml amphotericin B.

### Production and Preparation of Secreted Proteins for Comparative Secretome Analysis

To induce the secretion of PWN proteins, PWN populations (1 × 10^7^ nematodes per isolate) were soaked in 5-ml pine wood extracts on a 100-mm low attachment surface plate (EZ-BindShut dish; Iwaki) at 28°C for 16 h with agitation. The pine wood extract was prepared from nematode non-inoculated 3-year-old Japanese black pine, *P. thunbergii*, seedlings that is susceptible to PWN according to the method described in Shinya et al. (2013). The nematodes were then pelletized by centrifugation at 800 × *g* for 5 min at 25°C, and the supernatant was collected. Each resulting protein solution was desalted and buffer-exchanged in PD-10 columns (GE Healthcare) with 20 mM Tris-HCl (pH 8.0), in accordance with the manufacturer’s protocol. The solution containing secreted proteins was subsequently concentrated to 800 μl using an Amicon Ultra centrifugal filter (Millipore) with a 3-kDa cutoff, then subjected to two-dimensional high-performance liquid chromatography (2D-HPLC) analysis without adjusting the protein concentration. Three biological replicates were analyzed for each isolate.

### Protein Separation Using 2D-HPLC and Sodium Dodecyl Sulfate-Polyacrylamide Gel Electrophoresis

The extracted and desalted mass proteins were separated on a 2D-HPLC system using an ion-exchange column for the first dimension and a reverse-phase monolithic column for the second dimension (Morisaka et al., 2012). The 2D-HPLC system consisted of PU-712 and PU-714 pumps (GL Sciences), two trap columns (Nacalai Tesque), a 7725 injector (Rheodyne), a MU-701 UV detector (GL Sciences), and a 10-port valve MV-790 (GL Sciences). Raw chromatographic ASCII formatted data files were collected with EZChrom Elite software (GL Sciences). The secreted proteins were separated into six (F1–F6) fractions for the first-dimension extraction in ion-exchange mode. An anion-exchange column, NUCLEOSIL 4000-7 PEI (MACHEREY-NAGEL), was used with a flow rate of 0.8 ml/min for the ion-exchange mode for the first-dimension extraction. A gradient was provided by changing the mixing ratio of two eluents: A, 20 mM Tris/HCl (pH 8.2) and B, 20 mM Tris/HCl (pH 8.2) containing 1 M NaCl. The gradient involved six steps (F1–F6) with 0, 10, 20, 30, 50, or 100% B for 3 min. Each fraction was loaded into the second-dimension column through a 10-port valve and separated in reverse-phase mode. In reverse-phase mode, a wide-pore monolithic column [5 cm length, 2.3 mm inner diameter (ID)] was used with a flow rate of 1.0 or 1.5 ml/min. A gradient was provided by changing the mixing ratio of two eluents: C, 0.1% (v/v) trifluoroacetic acid and D, acetonitrile containing 0.1% (v/v) trifluoroacetic acid. The gradient initially contained 10% B, which was increased to 60% B for 5 or 3 min, then increased to 95% B to wash the column, and finally restored to the initial conditions and maintained for re-equilibration of the column. Because no protein was detected in fractions from 0 to 2 min in the preliminary test, the fractions including separated proteins were collected only from 2 min to 4.5 min at 30-s intervals for each ion-exchange mode fraction (F1–F6). In total, 30 fractions were collected during 2D-HPLC analysis. These fractions were dried by vacuum centrifugation and re-suspended in 10 μl of phosphate-buffered saline (pH 7.4). The re-suspended samples were mixed with an equal volume of 2× sodium dodecyl sulfate (SDS) sample buffer, then heated at 100°C for 3 min prior to loading onto a gel for subsequent SDS-polyacrylamide gel electrophoresis (SDS-PAGE) analysis.

Tricine SDS-PAGE and Laemmli SDS-PAGE systems were used. The fractions from 2 to 3 min (total of 12 fractions) were separated using a Tricine SDS-PAGE system with 15% Tris-tricine gels (e-PAGEL; ATTO) and a standard minislab PAGE apparatus (model AE-6500; ATTO) to separate low-molecular-weight proteins. Low-Range Rainbow Molecular Weight Markers (GE Healthcare) were used to determine the molecular masses of the proteins. In addition, the fractions from 3 to 4.5 min (total of 18 fractions) were separated using a Laemmli SDS-PAGE system with 5–20% gradient gels (e-PAGEL; ATTO). Full-Range Rainbow Molecular Weight Markers (GE Healthcare) were used to determine the molecular masses of the proteins. The proteins were silver-stained using a Sil-Best staining kit (Nacalai Tesque), in accordance with the manufacturer’s instructions.

### Gel Band Visualization and Relative Quantification

ImageJ software (National Institutes of Health, version 1.42) was used for relative quantitative densitometric analysis of gel band intensities. Eighty-nine selected bands with high repeatability were quantified based on their relative intensities. A relative fold change value was calculated for each gel band, compared with the expected value. The densitometry values for three independent replicates of each sample were subjected to Bartlett’s test to determine whether equal variances were present. When Bartlett’s test indicated that the group variances were equal, one-way analysis of variance (ANOVA) was performed, followed by Tukey’s multiple comparison *post hoc* test. When the group variances were unequal, the non-parametric Kruskal–Wallis multiple comparison *post hoc* test was used. A *P*-value of <0.01 was regarded as indicating statistical significance. Furthermore, fold changes in gel band intensities were calculated among isolates. Finally, proteins with gel bands with statistically significant fold change values that constituted >5-fold greater intensities for both virulent isolates (Ka4 and S10) relative to the avirulent isolates (C14-5 and OKD-1) were regarded as potential “virulence determinants” in this study. The log-transformed expression ratios of the 89 protein bands were subjected to hierarchical analysis with CLUSTER software and visualized in a clustergram with TreeView software.

### Tryptic In-Gel Digestion

In-gel digestion was performed as follows. Only the protein bands from Ka4 isolate were used for subsequent analysis. In accordance with the manufacturer’s protocol, protein bands with significant differences between samples were excised and destained using the Sil-Best Destain Kit (Nacalai Tesque). The destained gels were dehydrated in 100 μl of acetonitrile and completely dried via vacuum centrifugation. The excised gel pieces were incubated in freshly prepared 10 mM dithiothreitol/25 mM ammonium hydrogen carbonate (NH_4_HCO_3_) at 56°C for 1 h. The proteins were then alkylated in 55 mM iodoacetamide/25 mM NH_4_HCO_3_ in the dark for 45 min at room temperature. The gel pieces were subsequently dehydrated twice in 200 μl of 25 mM NH_4_HCO_3_ in 50% acetonitrile, then dried via vacuum centrifugation for 15 min. The samples were incubated in 20 μl of 50 mM NH_4_HCO_3_ containing 10 μg/ml trypsin (Promega Diagnostics) for 12 h at 37°C. Following enzymatic digestion, the resultant peptides were extracted twice with 50 μl and then 25 μl of 0.1% trifluoroacetic acid in 50% acetonitrile, and concentrated to 10–15 μl via vacuum centrifugation. Furthermore, the peptides were desalted using a MonoTip C18 (GL Sciences) and eluted with 0.1% trifluoroacetic acid in 50% acetonitrile.

### Nano Liquid Chromatography-Tandem Mass Spectrometry Analysis

Protein identification was performed using a liquid chromatography (LC; EASY-nLC II, Thermo Scientific)-mass spectrometry (MS; LTQ Velos orbitrap mass spectrometer, Thermo Scientific) system. First, 10 μl of proteolysis products was injected and separated via reverse-phase chromatography using a packed tip column (NTCC-360, 100 mm × 75 μm ID, Nikkyo Technos), at a flow rate of 300 nl/min. A gradient was achieved by changing the mixing ratio of two eluents: A, 0.1% (v/v) formic acid, and B, acetonitrile containing 0.1% (v/v) formic acid. The gradient was initiated with 5% B, increased to 40% B for 45 min, further increased to 95% B to wash the column, then returned to the initial condition, and maintained for re-equilibration. The separated analytes were detected on a mass spectrometer (with a full scan range of 300–2,000 m/z). The method was designed to automatically analyze the 10 ions with the highest intensity as observed in the MS scan for data-dependent acquisition. An ESI voltage of 1.5 kV was applied directly to the LC buffer distal to the chromatography column using a microtee. The ion-transfer tube temperature in the LTQ Velos ion trap was set at 250°C. The MS data were used for protein identification with Mascot software (Matrix Science), with a protein database built from *B. xylophilus* genome data (Kikuchi et al., 2011). MS/MS spectra were collected for *B. xylophilus* secreted proteins (Supplementary Data Sheets 1–4). The enzyme parameter was limited to full tryptic peptides with the maximum mis-cleavage default setting (carbamidomethylation of cysteines, ±6 ppm for precursor ions, ±0.6 Da for fragment ions). A concatenated forward-reverse database was constructed to calculate the *in situ* false discovery rate (Shaffer, 1995). An identification filtering criterion—1% false discovery rate—was used at the peptide level for each search.

### Gene Expression of Potential Virulence Determinants in Relation to Infection Process

Quantitative real-time polymerase chain reaction (PCR) analysis was used to quantify the gene expression levels of four potential virulence determinants: *Bx-lip1* (BUX.s00961.62), *Bx-GH30* (BUX.s00713.1066), *Bx-CAT1* (BUX.s01288.15), and *Bx-CAT2* (BUX.s00813.52).

#### Nematode Preparation and Inoculation Into Pine Seedlings

Four isolates of *B. xylophilus* were used in this experiment: two virulent isolates (Ka4 and S10) and two avirulent isolates (C14-5 and OKD-1). To investigate the gene expression levels of PWN target genes during *in vitro* culture and infection of pine seedlings, nematodes were propagated on the fungus *Botrytis cinerea* on potato dextrose agar at 25°C. After 10 days of incubation, nematodes were collected and 60,000 of each isolate was used to inoculate 3-year-old Japanese black pine seedlings following the method described by Shinya et al. (2010). At 3 and 7 days after inoculation (dai), the nematodes were extracted from woody pieces of inoculated seedlings using the Baermann funnel technique for 3 h, then washed five times in sterile water. The nematodes collected at three different time points (pre-inoculation, 3 dai, and 7 dai into pine seedlings) were prepared for total RNA extraction.

#### Preparation of Template cDNA via RNA Extraction and Reverse Transcription

For the extraction of total RNA, 5,000 nematodes of each isolate were used at each time point. Three biological replicates of each treatment were performed. Nematodes were ground to powder in liquid nitrogen using a mortar and pestle, and total RNA was extracted and purified with a RNeasy Plus Micro kit (Qiagen), in accordance with the manufacturer’s protocol. Reverse transcription (RT) was performed with 500 ng of total RNA from each treatment using a ReverTra Ace qPCR RT kit (Toyobo), in accordance with the manufacturer’s protocol. Transcribed cDNA was adjusted to 500 ng/μl and subjected to quantitative real-time PCR analysis.

#### Quantitative Real-Time PCR to Investigate Gene Expression of Potential Virulence Determinants During Host Infection

For each target gene, a primer pair was selected with Primer Express 3.0 software (Applied Biosystems) using the default settings for SYBR Green real-time PCR analyses. These primers were designed based on the genome sequence of *B. xylophilus* (Kikuchi et al., 2011). The nucleotide sequences of the primers are shown in Supplementary Table 1. The reaction mixture for real-time amplification included 12.5 μl of Power SYBR Green PCR Master Mix (Applied Biosystems), 1 μl of each primer (10 μM), 1 μl of template DNA, and 9.5 μl of distilled water. The real-time PCR conditions were as follows: an initial hot start at 95°C for 10 min, then 40 cycles of denaturation at 95°C for 15 s and annealing and extension at 60°C for 1 min. The reactions were performed on an Applied Biosystems 7500 real-time PCR system. The system software provided was used for standard curve analysis of the results. To correct for inter-sample variation, the β-tubulin gene (BUX.s01109.465) of the Ka4 isolate of *B. xylophilus* was used as an internal control. Three biological replicate samples were analyzed for each treatment–time combination with three technical replicates per sample. Gene expression levels were determined relative to that of the OKD-1 isolate on fungus (pre-inoculation). The statistical analysis was performed using a Tukey’s HSD test.

### Gateway Plasmid Construction for Leaf-Disk Assay With *N. benthamiana*

A leaf-disk assay based on transient overexpression in *N. benthamiana* was performed to allow functional screening of candidate virulence determinants of the PWN, in accordance with the method used by Kirino et al. (2020). Total RNA was extracted from mixed-stage nematodes and purified using a RNeasy Mini kit (Qiagen) for this assay, in accordance with the manufacturer’s protocol. RT was performed with 1 μg of total RNA using a PrimeScript^TM^ II 1st strand cDNA Synthesis kit (Takara Bio Inc.), in accordance with the manufacturer’s protocol. The transcribed cDNA was adjusted to a concentration of 200 ng/μl and subjected to PCR amplification. The sequences of *Bx-lip1*, *Bx-GH30*, *Bx-CAT1*, and *Bx-CAT2* were amplified from cDNA extracted from the Ka4 isolate of *B. xylophilus*. All PCR analyses were performed with Takara PrimeSTAR GXL DNA polymerase (Takara Bio Inc.), in accordance with the manufacturer’s protocol. The PCR components were as follows: 10 μl of 5X Prime STAR GXL buffer, 4 μl of dNTP mixture (2.5 mM each), 1 μl of forward primer (10 μM), 1 μl of reverse primer (10 μM), 1 μl of Prime STAR GXL polymerase, 1 μl of cDNA template (200 ng/μl), and 32 μl of H_2_O. The cycling conditions were as follows: 35 cycles at 98°C for 10 s, 55°C for 15 s, and 68°C for 60 s/kb. The primers were designed using Primer 3 Plus software^1^. The presence of secretory signal peptides (SPs) in the sequences of the associated genes was determined with the SignalP version 4.1 server. In this study, the constructs for each protein were prepared without a putative SP because the SPs were expected to be cleaved and discarded before secretion from the nematode stylet.

Gateway expression vectors were constructed with a BP reaction, using the Donor vector pDONR207, and with an LR reaction, using the Destination vector pUBC-GFP-dest, in accordance with the manufacturer’s specifications (Invitrogen). The target genes were amplified, and the stop codon was removed via PCR using primers containing the attB1 and attB2 sites. The amplification product was cloned into the pDONR 207 entry vector, then subcloned into the pUBC-GFP-Dest vector for C-terminal GFP fusion. The primers used in this analysis are listed in Supplementary Table 2.

### Transient Overexpression Assay of PWN Proteins in *N. benthamiana*

*Nicotiana benthamiana* plants were grown in a growth chamber at 20–30°C under a 16-h-light/8-h-dark cycle. For all assays, the sixth or seventh leaves of 4–6-week-old specimens were used. Expression vectors harboring each gene of the four candidates and GFP-only vector control were transformed into *Agrobacterium tumefaciens* strain C58 via electroporation. *A. tumefaciens* bacteria harboring expression vectors were grown in yeast extract beef medium containing 100 mg/l spectinomycin, 50 mg/l carbenicillin, and 25 mg/l rifampicin. Cultures of the *A. tumefaciens* suppressor strain P19 were grown at 28°C in yeast extract beef medium containing 50 mg/l kanamycin until an optical density at 600 nm of 0.5–0.7 was reached. Cells were harvested via centrifugation for 10 min at 3,500 rpm, then re-suspended to a final optical density at 600 nm of 0.3 in 10 mM MES buffer with 10 mM MgCl_2_ and 150 μM acetosyringone. Strains of *A. tumefaciens* were mixed in roughly equimolar quantities. The P19 suppressor strain was then added at approximately 25% of the total volume. The *A. tumefaciens* suspension was manually infiltrated into *N. benthamiana* leaves using a 1-ml syringe. Symptom development was visually monitored for 10 days after infiltration and categorized visually into two classes: cell death or no cell death (Kettles et al., 2017; Kirino et al., 2020). The proportions of spots indicating cell death were analyzed using Fisher’s exact test (^∗∗^*P* < 0.01). A total of 32 spots were examined (8 plants per gene and four spots per plant) for each candidate molecule. For the GFP control, a total of 120 spots were examined (30 plants and four spots per plant).

For co-localization analysis, an mCherry-labeled endoplasmic reticulum marker, ER-rk CD3–959, was included in a co-transformation procedure (Nelson et al., 2007). The third or fourth leaf of 3-week-old *N. benthamiana* plants was agro-infiltrated with GFP or GFP-fusion pathogenic candidate proteins with p19 and ER-rk CD3-959 in a 1:1:2 ratio. GFP or mCherry fluorescence in the epidermal cells of the abaxial leaf side was assessed at 3 days post-infiltration using an LSM 880 laser scanning microscope fitted with a 40× objective lens (Carl Zeiss). Pieces of the infiltrated leaf were sampled randomly from the infected area, and samples were mounted in 400 mM sucrose for observation via microscopy.

Western blot analysis was performed to validate the expression of Bx-GH-30-GFP fusion protein in *N. benthamiana* according to the method described in Kirino et al. (2020). Briefly, the gel was transferred to an Immun-Blot polyvinylidene fluoride membrane (Bio-Rad) using the *Trans*-Blot Turbo Transfer System (Bio-Rad) with *Trans*-Blot Turbo Midi Transfer Packs. The membrane was blocked in Tris-buffered saline with 0.05% Tween 20 with 1% skim milk powder. After the membrane had been blocked, it was incubated with a rabbit polyclonal anti-GFP antibody (MBL, diluted 1:10,000). Then, it was incubated with a polyclonal anti-IgG-HRP antibody (secondary antibody, diluted 1:10,000). After the membrane had been incubated with Western ECL Substrate (Bio-Rad), the image was visualized using an Image Quant LAS 4000 biomolecular imager (Fujifilm).

### *In situ* Hybridization in PWNs

*In situ* hybridization was performed to determine the localization of *Bx-GH30* transcript expression in PWNs, generally following the procedures of Espada et al. (2016). The gene sequences of *Bx-GH30* were PCR-amplified from cDNA extracted from *B. xylophilus*. In the first round of PCR, each fragment was amplified with Takara PrimeSTAR GXL DNA polymerase, in accordance with the protocol described above. The primers were designed using Primer 3 Plus software (primers listed in Supplementary Table 3). The resulting product was used as a template to synthesize ssDNA probes via linear PCR in a 30-μl reaction mixture [2.25 μl of digoxigenin–DNA labeling mix (Roche Diagnostics), 6 μl each of 10 μM reverse primer, 6 μl of PCR buffer, 0.6 μl of Prime STAR GXL polymerase, 2.25 μl of template, and 12.9 μl of H_2_O]. The cycling conditions were as follows: 95°C for 2 s, followed by 35 cycles of 95°C for 15 s, 55°C for 30 s, and 72°C for 90 s. The quality and yield of the reaction were assessed using electrophoresis in a 1.5% agarose gel. The remaining reaction volume was boiled for 10 min and immediately quenched on ice to denature the ssDNA probe.

Adult *B. xylophilus* nematodes cultured on the fungus *B. cinerea* were soaked in pine wood extract, in accordance with the protocol described by Shinya et al. (2013). They were then fixed for 18 h to few days at 4°C in 4 ml of 2% paraformaldehyde in M9 buffer, followed by 4 h in fixative at room temperature. The fixed nematodes were centrifuged and resuspended in 0.2% paraformaldehyde in M9 buffer, then divided into 2–5 fragments with a needle on a clean microscope slide. The nematode sections were washed twice in 1 ml of M9 buffer, then incubated at room temperature for 45 min in 0.5 mg/ml proteinase K (Life Technologies) in 500 μl of M9 buffer. After the nematodes had been washed in 1 ml of M9 buffer, they were pelleted, frozen at −20°C for 15 min, incubated for 30 s in 1 ml of cold methanol at −20°C, then incubated for 1 min in 1 ml of cold acetone. After the nematode sections had been pelleted at 13,000 rpm, they were rehydrated in distilled water.

The hybridization buffer contained 50% deionized formamide, 20% 20X SSC buffer, 1% 1X blocking reagent (10× blocking reagent: 10× maleic acid: diethyl pyrocarbonate-H_2_O at a 10:9:81 ratio), 2% SDS, 1% 100× Denhardt’s solution, 1 mM ethylenediaminetetraacetic acid, 0.2 mg/ml fish sperm DNA, and 0.15 mg/ml yeast tRNA. The rehydrated nematode sections were washed in 500 μl of hybridization buffer, then pre-hybridized in 150 μl of hybridization buffer at 50°C for 15 min with rotation. Pre-hybridized nematode sections were added to 20 μl of DNA-digoxigenin probe. The hybridization process was allowed to proceed for 18 h at 50°C with rotation. The nematode sections were washed three times and incubated for 15 min with 100 μl of 4× SSC (20% 20× SSC and 80% diethyl pyrocarbonate-H_2_O) at 50°C with rotation, washed three more times, then incubated for 20 min with 100 μl of 0.1× SSC/0.1% SDS at 50°C with rotation. The nematode sections were washed once in 100 μl of 1× maleic acid (10× maleic acid: diethyl pyrocarbonate-H_2_O at a 1:9 ratio) and incubated for 30 min with 100 μl of 1× blocking reagent at room temperature with rotation. The nematodes were then labeled for 2 h with 100 μl of alkaline phosphatase-conjugated anti-digoxigenin antibody fragments that had been diluted to 1:500 in blocking reagent. After the nematodes had been washed three more times and incubated with rotation for 15 min in 100 μl of 1× digoxigenin washing buffer (10× digoxigenin washing buffer: diethyl pyrocarbonate-H_2_O at a 1:9 ratio), they were stained overnight at 4°C in 100 μl of detection buffer with 0.34 μl of nitroblue tetrazolium and 0.35 μl X-phosphatase. Staining was terminated by two washes in diethyl pyrocarbonate-H_2_O with 0.1% Tween 20. The nematodes were then observed under differential interference contrast microscopy.

#### dsRNA Synthesis

Full-length cDNA clones encoding the *Bx-CAT1*, *Bx-CAT2*, and *Bx-GH30* genes of the Ka4 isolate were used as templates for RNA synthesis. Full-length cDNAs were synthesized from total RNA using a CapFishing full-length cDNA premix kit (Seegene), in accordance with the manufacturer’s protocol. The sequences of primers used in this experiment are shown in Supplementary Table 4. A dsRNA negative control was included in the experiments by amplifying a fragment from a gene encoding EGFP. The plasmid pYEX-GI3 was used as a template for the preparation of the negative control. PCR amplification was performed using the following cycle profile: 94°C for 2 min, followed by 35 cycles of 98°C for 10 s, 56°C for 30 s, and 68°C for 1 min, and a final step of 68°C for 7 min. The PCR products were purified using a MiniElute PCR purification kit (Qiagen), and the quality and yield of the reactions were checked using agarose gel electrophoresis. Sense and antisense RNAs were synthesized in a single *in vitro* reaction using a MEGAscript RNAi kit (Ambion), in accordance with the manufacturer’s instructions. The quality of the generated dsRNA was checked using agarose gel electrophoresis and quantified with a NanoDrop 1000 spectrophotometer. Each dsRNA product was adjusted to a final concentration of 8 μg/μl in nuclease-free water and stored at −80°C.

#### dsRNA Treatment

RNA interference (RNAi) soaking treatment was performed generally in accordance with the method of Cheng et al. (2010). A mixed culture of the propagative forms was prepared by culturing on the fungus *B. cinerea*, which was grown on potato dextrose agar medium at 25°C. After 10 days of incubation, eggs were collected and the development of nematodes was synchronized by allowing the J2 to hatch in the absence of food. Then, only J2 and J3 PWNs were collected as described in Shinya et al. (2009). The soaking protocol involved RNA dissolution in M9 soaking buffer (43 mM Na_2_HPO_4_, 22 mM KH_2_P0_4_, 2 mM NaCl, and 4.6 mM NH_4_Cl). A 20-μl aliquot of a suspension containing 20,000 J2 and J3 nematodes was mixed with 50 μl of dsRNA solution (final concentration of 4 μg/μl) with 50 mM octopamine (Sigma). The PWN suspensions were shaken lightly (130 rpm) in a rotary shaker for 24 h at 25°C. Control treatments included 50 μl of distilled water, instead of dsRNA solution.

### Transcript Analysis Using Quantitative Real-Time PCR

For each silencing assay, 5,000 nematodes were collected after soaking in dsRNA solution (4 μg/μl) or control solution. The methods of total RNA extraction and quantitative real-time PCR analysis were identical to those described above.

### Effect of RNAi Treatment on *B. xylophilus* Reproduction on Gray Mold

The feed fungus *B. cinerea* was initially cultured at 25°C for approximately 6 days on potato dextrose agar medium in a 35-mm plate. A 10-μl suspension containing 100 nematodes, collected after soaking in a dsRNA solution (4 μg/μl) or control solution, was inoculated on the fungal mat and incubated at 25°C. After 10 days, nematodes were collected using the Baermann funnel method (25°C for 24 h) and counted under a stereomicroscope. All experiments were performed with eight biological replicates. The number of PWNs in the eight replicate samples was subjected to statistical analysis. Multiple comparisons were performed with one-way ANOVA. When the ANOVA revealed a significant difference (*P* < 0.05) among groups, Tukey’s honestly significant difference (HSD) test was performed.

## Results

### Quantitative Densitometric Analysis Based on Protein Band Intensity

**FIGURE 1:**
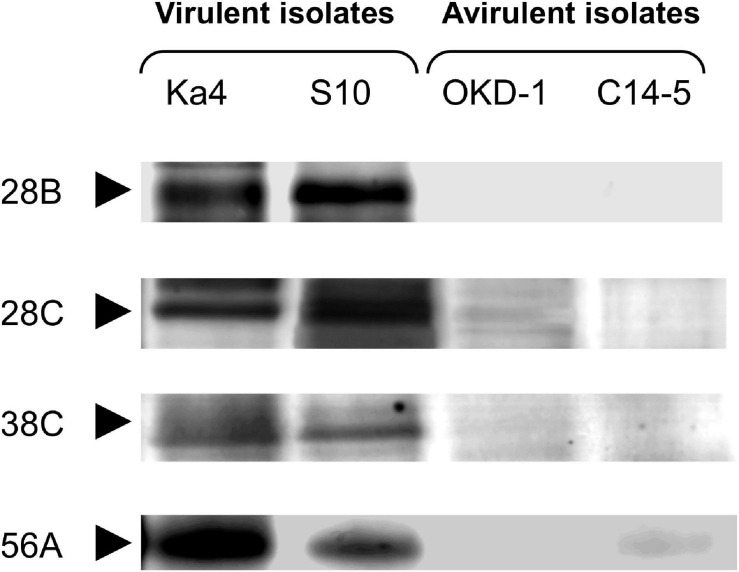
Comparative analysis with sodium dodecyl sulfate-polyacrylamide gel electrophoresis (SDS-PAGE) of *Bursaphelenchus xylophilus* secreted proteins derived from two-dimensional high-performance liquid chromatography (2D-HPLC) fractions. Each fraction was run in a sequential manner. Four protein bands that were significantly different from the others (*P* < 0.05; Tukey’s HSD test) with >5-fold greater intensities in both virulent isolates (Ka4 and S10), compared with avirulent isolates (C14-5 and OKD-1), are shown.

**FIGURE 2:**
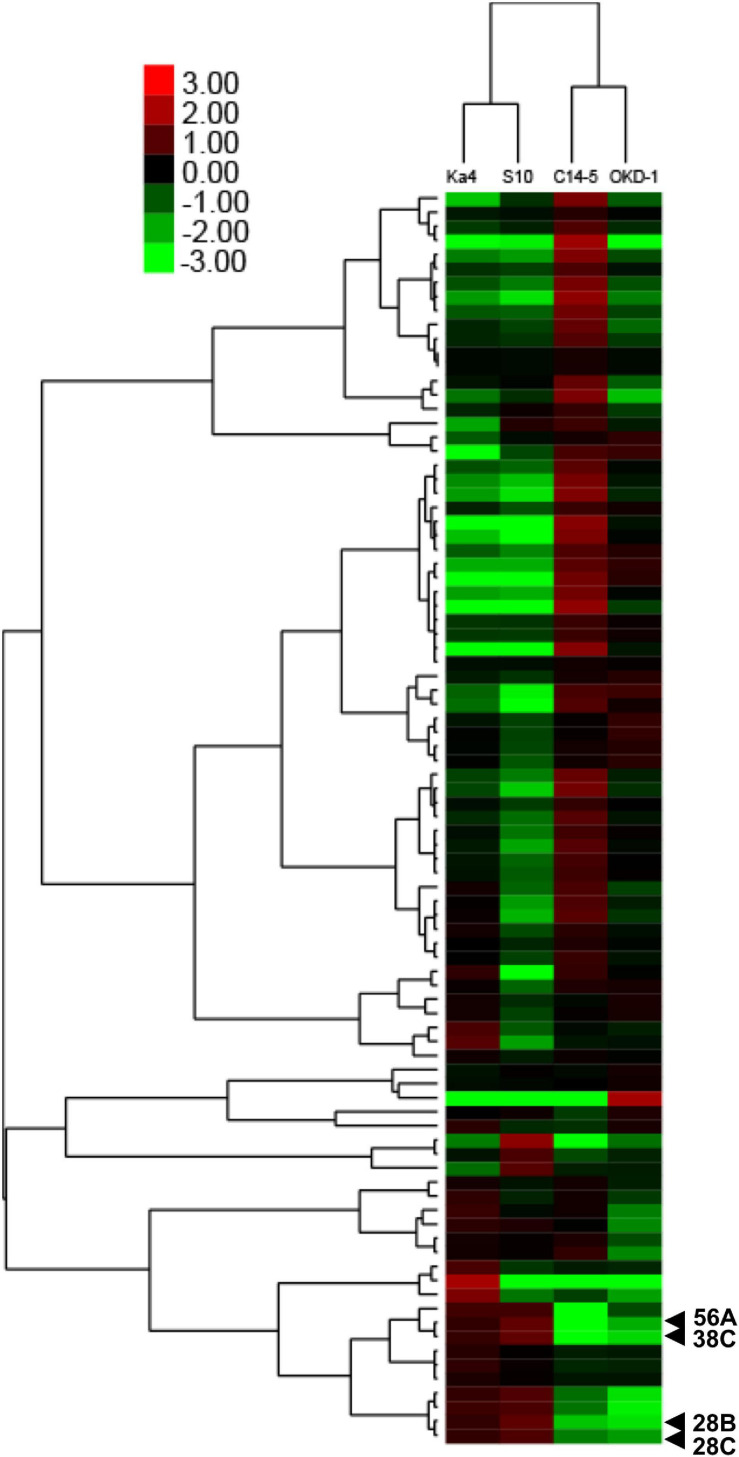
Two-way hierarchical clustering analysis of different amounts of secreted proteins based on relative quantitative densitometric analysis of gel band intensities using ImageJ software. The *x*-axis indicates isolates of *B. xylophilus* and the *y*-axis indicates the mean values of log_2_ ratios of relative fold changes calculated for each gel band, compared with the expected value. Four protein bands that were significantly different from the others (*P* < 0.05; Tukey’s HSD test) with >5-fold greater intensities in both virulent isolates (Ka4 and S10), compared with avirulent isolates (C14-5 and OKD-1), are indicated.

Analyses using 2D-HPLC and SDS-PAGE revealed 89 protein bands with high repeatability. These gel bands were subjected to relative quantitative densitometric analysis using ImageJ software (Figure 1 and Supplementary Figure 1). Subsequently, hierarchical clustering analysis was performed for these 89 protein bands, revealing four potential virulence determinants—bands 28B, 28C, 38C, and 56A (Figure 2), with molecular masses of 32.5, 59, 31.5, and 23.5 kDa, respectively. Clustering analysis also showed that the protein expression profiles differed between virulent and avirulent isolates of *B. xylophilus*.

### Identification of Potential Virulence Determinants Using LC-MS/MS Analysis

To characterize the potential virulence determinants identified in the above screening, nanoLC-MS/MS analysis was performed using a *B. xylophilus* genome database. The potential virulence determinants are listed in Table 1. They were identified as class 3 lipase (BUX.s00961.62), glycosyl hydrolase family 30 (BUX.s00713.1066), cathepsin L (BUX.s01288.15), and cathepsin L1 (BUX.s00813.52). Henceforth, they are referred to as Bx-lip1, Bx-GH30, Bx-CAT1, and Bx-CAT2, respectively. All of these proteins were previously identified in the *B. xylophilus* secretome, and these were at least sixfold more abundant than the whole *B. xylophilus* proteins (Shinya et al., 2013).

**TABLE 1:** List of potential virulence determinants identified via liquid chromatography-tandem mass spectrometry analysis.

Band no.	Protein ID	Annotation	Theo. MM (kDa)	Coverage (%)	Relative expression* (fold change)
28B	BUX.s00961.62	Class 3 lipase	32.5	21	10.2
28C	BUX.s00713.1066	Glycosyl hydrolase family 30	58.5	19	5.3
38C	BUX.s01288.15	Cathepsin L	26.5	10	15.1
56A	BUX.s00813.52	Cathepsin L	44.5	16	10.2

*Relative expression indicates mean fold change of the expression level in virulent isolates (Ka4 and S10) relative to that in avirulent isolates (C14-5 and OKD-1).

### Gene Expression of Potential Virulence Determinants During Host Infection

**FIGURE 3:**
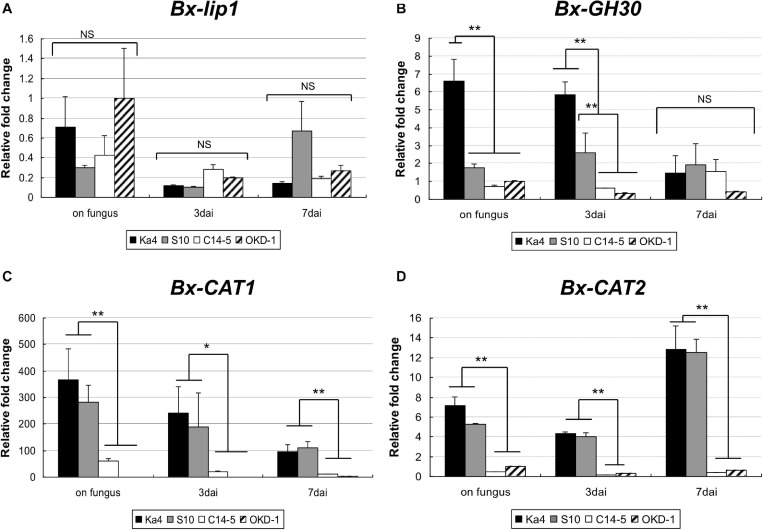
Temporal gene expression levels of **(A)** *Bx-lip1*, **(B)** *Bx-GH30*, **(C)** *Bx-CAT1*, and **(D)** *Bx-CAT2* in four isolates of *B. xylophilus* as evaluated using real-time polymerase chain reaction (PCR). Gene expression levels were determined relative to that of the OKD-1 isolate on fungus (pre-inoculation). Each bar represents the mean ± SD of three biological replicates (*P < 0.05, **P < 0.01, Tukey’s HSD test).

Using real-time RT-PCR, the transcript levels of the four potential virulence determinants of *B. xylophilus* were measured at three time points: pre-inoculation (on fungus culture) and 3 and 7 dai into host pines (Figure 3). The transcript levels of *Bx-CAT1*, *BxCAT-2*, and *Bx-GH30* were significantly higher in virulent isolates than in avirulent isolates at pre-inoculation and 3 dai. Only the *Bx-GH30* transcript level declined in virulent isolates at 7 dai, such that no significant difference was observed between virulent and avirulent isolates at 7 dai. The *Bx-CAT1* transcript level was always more than 100-fold greater in virulent isolates than in avirulent isolates. Temporal analysis showed that the transcript levels of *Bx-GH30* and *Bx-CAT1* tended to be high during the pre-infection and early infection periods (3 dai), and the transcript level of *Bx-CAT2* was higher in the later infection period (7 dai). Only the *Bx-lip1* transcript level was not consistent with the results of comparative proteome analysis, in which the amount of Bx-lip1 protein was significantly greater in virulent isolates than in avirulent isolates. At the transcript level, no significant differences were observed in the expression of *Bx-lip1* between virulent and avirulent isolates at all three time points (Figure 3A) (*P* > 0.01, Tukey’s HSD test).

### Bx-GH30 Influence on Plant Cell Death

**FIGURE 4:**
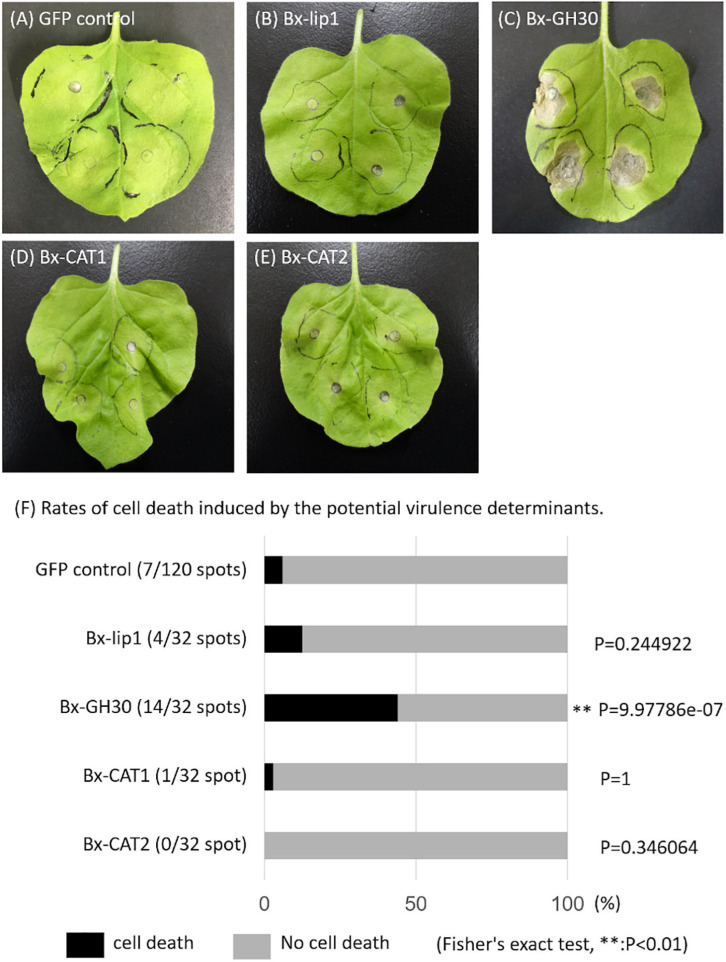
Symptoms and rates of cell death induced by potential virulence determinants. Leaves of *Nicotiana benthamiana* were infiltrated with *Agrobacterium tumefaciens* strains to express candidate *B. xylophilus* proteins. Images were captured at 10 days after infiltration: **(A)** the GFP-only vector control, **(B)** Bx-lip1, **(C)** Bx-GH30, **(D)** Bx-CAT1, and **(E)** Bx-CAT2. **(F)** The rates of cell death induced by candidate molecules are presented graphically. The degree of symptoms after infiltration was monitored visually and categorized into two classes: cell death (black bar) and no cell death (gray bar). The proportions of cell death in parentheses were statistically analyzed using Fisher’s exact test (^∗∗^*P* < 0.01). Asterisks indicate a significant increase in cell death induced by one of the four candidate pathogenic proteins, compared with the GFP-only vector control.

Using a pUBQ10 promoter, transgenic *N. benthamiana* lines were generated expressing each of the four potential virulence determinant proteins, and their contributions to tobacco plant cell death were investigated (Figure 4). The presence of SPs within the sequences of the associated genes was determined using the SignalP server (version 4.1). Predicted SPs in the sequences of Bx-lip1, Bx-GH30, and Bx-CAT2 were excised. In the sequence of Bx-CAT1, an SP was not predicted. Bx-GH30 induced significant cell death relative to the GFP-only control (*P* < 0.01). No significant cell death relative to the GFP-only control was observed in treatments with the other potential virulence determinants: Bx-lip1, Bx-CAT1, and Bx-CAT2.

### Bx-GH30 Localization in Plants

**FIGURE 5:**
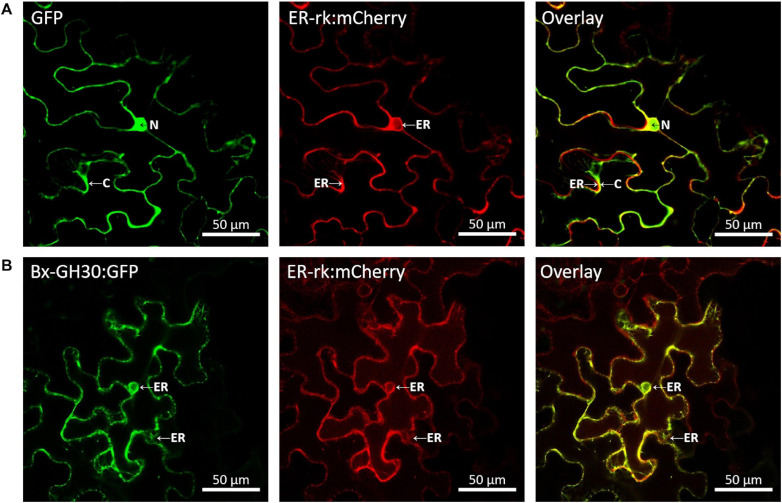
Localization of Bx-GH30 in plant cells. Confocal images of GFP **(A)** and Bx-GH30 **(B)** infiltrated into *N. benthamiana* leaves, together with an mCherry-labeled endoplasmic reticulum marker (ER-rk CD3-959). For confocal laser scanning microscopy, samples were taken at 3 days post-inoculation, and fluorescent channels were scanned sequentially. GFP fluorescence is shown in green (left panel) and mCherry fluorescence is shown in red (middle panel). The overlay of green and red signals appears yellow (right panel). N, nucleus; C, cytoplasm; ER, endoplasmic reticulum. Scale bar = 50 μm.

To determine its subcellular localization, Bx-GH30 was fused to the gene encoding GFP, then transfected into *N. benthamiana* with an mCherry-labeled endoplasmic reticulum marker (ER-rk CD3–959). The transiently expressed GFP fusion protein and mCherry-labeled endoplasmic reticulum marker in tobacco leaves was visualized using confocal microscopy (Figure 5). The GFP signal of Bx-GH30 was co-localized with the endoplasmic reticulum marker. By contrast, the GFP signal of the vector control was present in both the plant cytoplasm and nucleus. In addition to the observation by fluorescence microscopy, the expression of Bx-GH-30-GFP fusion protein was validated by Western blot analysis using anti-GFP polyclonal antibodies. As a result, the 83 kDa protein band was detected in the Bx-GH30 sample and was consistent with the predicted molecular mass (Supplementary Figure 2).

### *Bx-GH30* mRNA Expression in Adult *B. xylophilus*

*In situ* mRNA hybridization was used to investigate the spatial expression patterns of *Bx-GH30* in adult PWNs soaked in pine wood extract. Digoxigenin-labeled antisense probes generated from the cellulase gene (*Bx-ENG-1*; reportedly expressed in esophageal gland cells) were used as a positive control (Kikuchi et al., 2004). The *Bx-ENG-1* signal was detected specifically in esophageal gland cells (Supplementary Figure 3A). Although no clear localization was obtained in a specific area, a diffuse signal was observed for *Bx-GH30* around esophageal gland cells (Supplementary Figure 3C). Similar signal patterns were detected in both female and male nematodes. No hybridization with control sense cDNA probes was observed in the nematodes (Supplementary Figures 3B,D).

### Evaluation of RNAi Efficiency Using Real-Time PCR Analyses

Quantitative real-time RT-PCR was performed to confirm the efficiency of gene silencing by RNAi. The ds*Bx-GH30* and ds*Bx-CAT2* treatments had some effects on *Bx-GH30* and *Bx-CAT2* transcript levels, respectively (Supplementary Figure 4). Taking the mRNA transcript level of the control (treated with non-dsRNA) as 100%, the mean transcript levels of *Bx-GH30* and *Bx-CAT2* were 66 and 49%, respectively (*P* < 0.01, Student’s *t*-test). No significant effect on *Bx-CAT1* transcript levels was observed after RNAi treatment, with a mean transcript level of 82% (*P* > 0.01, Student’s *t*-test). The negative control, ds*EGFP*, caused no significant changes in transcript levels of *Bx-GH30*, *Bx-CAT*, or *Bx-CAT2*, indicating that the gene-silencing effect observed in this experiment was sequence-specific.

### Effects of RNAi on *B. xylophilus* Reproduction on Gray Mold

**FIGURE 6:**
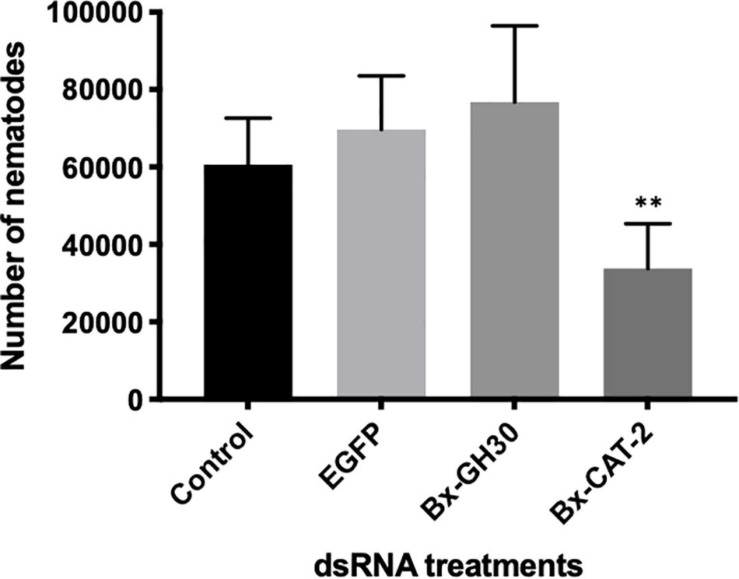
Effects of RNA interference (RNAi) treatment on *B. xylophilus* reproductive ability. Each bar represents the mean ± SD of eight replicates (^∗∗^*P* < 0.01, Student’s *t*-test).

To investigate the effects of RNAi on *B. xylophilus* reproductive ability, nematodes were counted after treatment with each dsRNA or M9 buffer (control) over 10 days of culture at 25°C on gray mold (Figure 6). As a negative control, dsRNA for non-endogenous *EGFP* was used to assess dsRNA toxicity, revealing no toxicity. Nematodes treated with ds*Bx-CAT2* propagated significantly less than did control nematodes (*P* < 0.01, Student’s *t*-test). However, no significant reductions in nematode number were observed after treatment with ds*Bx-GH30*, or ds*EGFP*, compared with the control (*P* > 0.01, Student’s *t*-test). *Bx-CAT1* was omitted from this portion of the study because no significant effect on *Bx-CAT1* transcript levels was observed after RNAi treatment (Supplementary Figure 4).

## Discussion

In the present study, a comparative secretome analysis of four isolates of *B. xylophilus* with distinct degrees of virulence against host pines was performed using 2D-HPLC and SDS-PAGE, combined with LC-MS/MS analysis. We initially targeted 89 protein bands with high reproducibility and quantified them on the basis of protein band intensities. We identified four proteins that were highly expressed in two virulent isolates: class 3 lipase (Bx-lip1), glycosyl hydrolase family 30 (Bx-GH30), cathepsin L (Bx-CAT1), and cathepsin L (Bx-CAT2) (Table 1). All of these were also identified in our previous comprehensive analysis of the *B. xylophilus* secretome (Shinya et al., 2013). In the present study, a 3D-protein separation system was used for comparative and semi-quantitative proteome analysis. In recent decades, label-based protein quantification techniques such as iTRAQ and SILAC have been used for comparative proteomics (Ong et al., 2002; Ross et al., 2004). Although these label-based methods are generally considered more sensitive and comprehensive compared to the gel-based methods used in our study, we presume that we have identified the major differentially secreted proteins between virulent and avirulent isolates. To validate quantitative differences in these four potential virulence determinants between virulence groups at the transcript level, quantitative RT-PCR was carried out at three time points (pre-inoculation, 3 dai, and 7 dai). The transcript levels of *Bx-CAT1*, *BxCAT-2*, and *Bx-GH30* were significantly higher in virulent isolates than in avirulent isolates at pre-inoculation and 3 dai, although the *Bx-GH30* transcript level in virulent isolates declined by 7 dai (Figure 3). These findings were consistent with the results of quantitative analysis at the protein level. The expression levels of *Bx-CAT1* and *Bx-GH30* genes were upregulated in virulent isolates during fungus feeding and the early phase of infection. Furthermore, *BxCAT-2* was upregulated during the late phase of infection. Only *Bx-lip1* exhibited a gene expression pattern that differed from the protein dynamics results. This implies that the amount of Bx-lip1 protein secreted by *B. xylophilus* was regulated in a post-transcriptional manner, and the levels of the other three proteins were mainly regulated at the transcript level. The gene expression level of *Bx-CAT1* was higher in virulent isolates than in avirulent isolates at all time points examined, but the highest expression was observed when PWNs were cultured on fungus. In *Bx-CAT2*, gene expression levels were lowest at 3 dai and higher during growth on fungus and at 7 dai in the pine seedlings. This suggests that *Bx-CAT1* and *Bx-CAT2* are enzymes that aid in fungus digestion and nutrient uptake. It remains unclear why the gene expression of *Bx-CAT2* was downregulated after PWNs entered the pine seedlings, although the PWNs might have migrated rapidly inside the tree after invasion (without a feeding phase). Ekino et al. (2020) demonstrated phenotypic plasticity in PWNs and revealed differences in ultrastructure between phytophagous and mycetophagous phases. In particular, atrophied intestinal microvilli were observed in the phytophagous phase, compared with the mycetophagous phase, suggesting that the phytophagous phase is a stage of active migration within the living pine tree, instead of feeding. Virulent isolates (e.g., Ka4 and S10) reportedly exhibited more robust reproductive ability, compared with avirulent isolates (e.g., C14-5 and OKD-1), when cultured on the fungus *B. cinerea* (Aikawa and Kikuchi, 2007; Shinya et al., 2012). It is likely that Bx-CAT1 and Bx-CAT2 are responsible for food digestion and contribute to reproductive ability. Among the four proteins differentially secreted between virulent and avirulent isolates, Bx-GH30 exhibited a unique gene expression pattern. In particular, significantly greater gene expression was observed in virulent isolates only at 3 dai.

To investigate the molecular functions of Bx-lip1, Bx-CAT1, Bx-CAT2, and Bx-GH30 during plant infection, we conducted an *in planta* functional analysis using *N. benthamiana* to screen for molecules that induced hypersensitive cell death in tobacco. Notably, Bx-GH30 induced significant cell death in *N. benthamiana*, relative to the GFP-only control (Figure 4). The GH30 family contains glycoside hydrolases with several known enzyme activities, such as β-glucosylceramidase and β-1,6-glucanase, and divided into largely two groups, distinguished by levels of sequence identity (St John et al., 2010). The amino acid sequence of Bx-GH30 secreted by *B. xylophilus* shows high similarity with the enzymes characterizing glucosylceramidase. β-glucosylceramidase hydrolyzes β-glucoside from the glycolipid glucosylceramide and from glucose-conjugated sphingolipids. Glucosylceramides are major components of the endomembrane system and plasma membrane in most eukaryotic cells. Although the molecular functions of glucosylceramides are not well-characterized, ceramides and sphingolipids have been identified as lipid signaling molecules (Hannun and Obeid, 2008) and are involved in programmed plant cell death associated with plant defense (Dunn et al., 2004; Saucedo-García et al., 2011; Berkey et al., 2012). GH30 also has a β-1,6-glucanase function. However, because β-1,6-glucans are major yeast cell-wall components, GH30 having a β-1,6-glucanase is unlikely to be involved in the induction of plant cell death. To verify the actual molecular function of Bx-GH30, a recombinant Bx-GH30 protein should be designed, which will allow examination of its function as an enzyme.

To determine the subcellular localization of Bx-GH30 in tobacco, its localization was observed using a GFP fusion protein and a mCherry-labeled endoplasmic reticulum marker (ER-rk CD3–959). Notably, Bx-GH30 was localized in the endoplasmic reticulum (Figure 5). Conversely, the GFP signal of the vector control was present in both the plant cytoplasm and nucleus. Given that an endoplasmic reticulum stress-inducible protein was upregulated in PWN-infested *Pinus pinaster* (a pine species very susceptible to PWNs), compared with PWN-infested *Pinus pinea* (a less susceptible pine species) (Santos et al., 2012), Bx-GH30 localized in the endoplasmic reticulum may induce hypersensitive cell death in pines. During the process of pine wilt disease, a hypersensitive reaction is accompanied by the leakage of abnormal oleophilic substances from parenchymal cells, resulting in blockage of the water conducting system (Futai, 2013). Bx-GH30 may play an important role in pine wilt disease as a trigger to induce a hypersensitive reaction. In addition to clarifying the enzymatic activity of Bx-GH30 as mentioned above, further studies to investigate Bx-GH30 localization in host pine trees are needed in the future. In this study, we also performed *in situ* hybridization analysis and found that Bx-GH30 was expressed around esophageal gland cells in PWNs (Supplementary Figure 3). The pharyngeal gland cells are the source of most nematode effectors (e.g., Haegeman et al., 2012). Therefore, Bx-GH30 may be produced in the pharyngeal gland cells and secreted from the nematode stylet into the plant apoplast or plant cells. Our findings demonstrate that Bx-GH30 causes cell death in tobacco, but its mechanisms are unclear. Further detailed studies are needed to determine how Bx-GH30 causes plant cell death and elucidate its specific role in pine wilt disease.

RNAi was used to examine the functions of potential virulence determinant genes during reproduction on gray mold. *Bx-lip1* was omitted from this portion of the study because our above results suggested that the amount of Bx-lip1 protein was regulated in a post-transcriptional manner. Furthermore, no significant effect on *Bx-CAT1* transcript levels was observed after RNAi treatment. In the analysis of *B. xylophilus* reproductive ability, silencing of the *Bx-CAT2* gene resulted in a reduction in the number of PWNs cultured on the fungal mat of *B. cinerea*. By contrast, no significant effect was observed after treatment with ds*Bx-GH30*, compared with the control. These results suggest that Bx-CAT2 is responsible for reproduction on gray mold, but that Bx-GH30 is not, indicating that only Bx-GH30 plays an important role in the induction of cell death within the plant. It has been reported that RNAi targeting of the *GH45* gene in *B. xylophilus* caused a decrease in reproductive ability (Cheng et al., 2010; Ma et al., 2011). Ma et al. (2011) also reported that silencing of the *GH45* gene resulted in reduced fungal-feeding ability. Because GH45 protein exhibits cellulose-degradation activity, silencing of the *GH45* gene may reduce the fungal cell wall degradation ability, thus impacting the reproductive ability of PWNs (Ma et al., 2011). However, in the present study, no clear difference was observed in the rate of fungal feeding among treatments (data not shown). Therefore, *Bx-CAT2* may be involved in nutrient uptake, and the silencing of this gene might have caused a substantial decrease in *B. xylophilus* reproductive ability. A clear difference in reproductive ability on the fungal mat has been reported between virulent and avirulent isolates (Aikawa and Kikuchi, 2007; Shinya et al., 2012). Palomares-Rius et al. (2015) suggested that avirulent isolates of *B. xylophilus* are likely to be the lack of activities of digestive proteases due to the loss of function mutations based on a comparative genomics between virulent and avirulent isolates. Bx-CAT2 is presumably responsible for this difference in reproductive ability on fungus because it is secreted more abundantly in virulent isolates than in avirulent isolates. In addition to differences in reproductive ability, remarkable differences have been reported between virulent and avirulent isolates in other biological traits, such as the ability to disperse in host tissues (Ichihara et al., 2000) and to tolerate oxidative stress (Vicente et al., 2015). Since it has been suggested that the ability of *B. xylophilus* to avoid and/or tolerate plant defense response is a key element in the process of pine wilt (Shinya et al., 2013; Vicente et al., 2015; Ding et al., 2016; Filipiak et al., 2020), the potential virulence determinants found in this study may have different functions involved in these traits. RNAi is a valuable tool for studying the biological function of genes because it causes sequence-specific degradation of mRNA mediated by homologous dsRNA, which inhibits gene function in a post-transcriptional manner (Hammond et al., 2001). However, the effects of RNAi are transient and weak in most nematodes other than *Caenorhabditis elegans* (Geldhof et al., 2007; Knox et al., 2007; Viney and Thompson, 2008), and the efficiency of this approach is strongly influenced by the type of target gene. In contrast to analyses of *C. elegans*, the reduction of gene expression was limited in our study. Moreover, the effect was transient and could not be inherited. Because of the limited effect of RNAi in *B. xylophilus*, a pathogenicity test using RNAi could not be conducted. Thus, the development of an alternative approach for further analysis is urgently needed to more comprehensively understand the molecular basis of *B. xylophilus* parasitism, and the functional analysis using *N. benthamiana* employed in this study will be a powerful tool in future analyses of pine wilt disease.

This study demonstrated that Bx-GH30, which is abundantly secreted by virulent isolates of *B. xylophilus*, can induce cell death in *N. benthamiana*. Bx-GH30 is a virulence-determining molecule among *B. xylophilus* isolates and is presumably an effector that causes cell death in host pines. Furthermore, Bx-CAT2 was involved in nutrient uptake during fungal feeding and contributed to differences in reproductive ability on fungus between virulent and avirulent isolates. To determine the molecular functions of two other potential virulence determinants, *Bx-lip1* and *Bx-CAT1*, a gene-knockout method must be established in *B. xylophilus*.

## Data Availability Statement

The original contributions presented in the study are included in the article/Supplementary Material, further inquiries can be directed to the corresponding author.

## Author Contributions

RS and HM designed the study. RS, HK, and HM performed the research and analyzed the data. RS, HK, HM, YT-K, KF, and MU wrote the manuscript. All authors contributed to the article and approved the submitted version.

## Conflict of Interest

The authors declare that the research was conducted in the absence of any commercial or financial relationships that could be construed as a potential conflict of interest.

## Footnotes

1 http://www.bioinformatics.nl/cgi-bin/primer3plus/primer3plus.cgi

## Supplementary Material

Supplementary Figure 2 SDS-PAGE comparative analysis of *B. xylophilus* proteins derived from 2D-HPLC fractions. Each fraction was run in a sequential manner for the four isolates of *B. xylophilus* (Ka4, S10, C14-5, and OKD-1).

Supplementary Figure 2 Bands of Bx-GH30 on Western blot membranes. GFP-only vector control protein and Bx-GH-30-GFP fusion protein expressed in *N. benthamiana* were detected with anti-GFP antibodies. The GFP control band is approximately 27 kDa. Bx-GH30 band is approximately 83 kDa.

Supplementary Figure 3 mRNA localization of *Bx-GH30* in nematodes. The localization of *Bx-ENG-1* **(A)** as a positive control for gene expression in pharyngeal gland cells, after *in situ* hybridization. Localization of transcripts encoding *Bx-GH30* of the pine wood nematode around pharyngeal glands, after *in situ* hybridization **(C)**. No signals were produced using the sense probes for *Bx-ENG-1* **(B)** and *Bx-GH30* **(D)**.

Supplementary Figure 4 mRNA expression levels of *Bx-GH30* **(A)**, *Bx-CAT1* **(B)**, and *Bx-CAT2* **(C)** genes in *B. xylophilus* after soaking in target dsRNA solution and EGFP dsRNA solution (a non-endogenous gene), relative to the control level (non-dsRNA treatment). Each bar represents the mean ± SD of three biological replicates. The expression level in the control was regarded as 1.0. Different letters indicate significant differences between genes (*P* < 0.01, Student’s *t*-test).

Supplementary Table 1 Primers used in real-time PCR analyses to quantify transcript levels.

Supplementary Table 2 List of primers used in Gateway vector construction.

Supplementary Table 3 List of primers used in *in situ* hybridization.

Supplementary Table 4 Primers used to prepare dsRNA for nematode soaking experiments.

Supplementary Data Sheet 1 MS/MS spectra for the protein band 28B.

Supplementary Data Sheet 2 MS/MS spectra for the protein band 28C.

Supplementary Data Sheet 3 MS/MS spectra for the protein band 38C.

Supplementary Data Sheet 4 MS/MS spectra for the protein band 56A.

## References

[B1] Abelleira A. Picoaga A. Mansilla J. P. Aguin O. (2011). Detection of *Bursaphelenchus Xylophilus*, causal agent of pine wilt disease on *Pinus pinaster* in northwestern Spain. *Plant Dis.* 95 776–776. 10.1094/PDIS-12-10-0902 30731923

[B2] Aikawa T. Kikuchi T. (2007). Estimation of virulence of *Bursaphelenchus xylophilus* (Nematoda: Aphelenchoididae) based on its reproductive ability. *Nematology* 9 371–377. 10.1163/156854107781352007

[B3] Aikawa T. Kikuchi T. Kosaka H. (2003). Demonstration of interbreeding between virulent and avirulent populations of *Bursaphelenchus xylophilus* (Nematoda: Aphelenchoididae) by PCR-RFLP method. *Appl. Entomol. Zool.* 38 565–569. 10.1303/aez.2003.565

[B4] Akiba M. (2006). Diversity of pathogenicity and virulence in the pinewood nematode, *Bursaphelenchus xylophilus*. *J. Jpn. For. Soc.* 88 383–391.

[B5] Baermann G. (1917). The conditions of spread of ankylostomiasis by ground infection and its control. *Geneesk. Tijdschr. v. Nederl-Indie.* 57 131–137.

[B6] Berkey R. Bendigeri D. Xiao S. (2012). Sphingolipids and plant defense/disease: the “death” connection and beyond. *Front. Plant Sci.* 3:68. 10.3389/fpls.2012.00068 22639658 PMC3355615

[B7] Cardoso J. Anjo S. Fonseca L. Egas C. Manadas B. Abrantes I. (2016). *Bursaphelenchus xylophilus* and *B. mucronatus* secretomes: a comparative proteomic analysis. *Sci. Rep.* 6:39007. 10.1038/srep39007 27941947 PMC5150578

[B8] Cheng X. Y. Dai S. M. Xiao L. Xie B. Y. (2010). Influence of cellulase gene knockdown by dsRNA interference on the development and reproduction of the pine wood nematode, *Bursaphelenchus xylophilus*. *Nematology* 12 225–233. 10.1163/138855409X12469541205044

[B9] Ding X. Ye J. Lin S. Wu X. Li D. Nian B. (2016). Deciphering the molecular variations of pine wood nematode *Bursaphelenchus xylophilus* with different virulence. *PLoS One* 11:e0156040. 10.1371/journal.pone.0156040 27224277 PMC4880305

[B10] Dunn T. M. Lynch D. V. Michaelson L. V. Napier J. A. (2004). A post-genomic approach to understanding sphingolipid metabolism in *Arabidopsis thaliana*. *Ann. Bot.* 93 483–497. 10.1093/aob/mch071 15037448 PMC4242313

[B11] Ekino T. Kirino H. Kanzaki N. Shinya R. (2020). Ultrastructural plasticity in the plant-parasitic nematode, *Bursaphelenchus xylophilus*. *Sci. Rep.* 10:11576. 10.1038/s41598-020-68503-3 32665657 PMC7360551

[B12] Espada M. Akker S. Maier T. Vijayapalani P. Baum T. Mota M. (2018). STATAWAARS: a promoter motif associated with spatial expression in the major effector-producing tissues of the plant-parasitic nematode *Bursaphelenchus xylophilus*. *BMC Genomics* 19:553. 10.1186/s12864-018-4908-2 30053797 PMC6062891

[B13] Espada M. Silva A. C. Akker S. E. van den Cock P. J. A. Mota M. Jones J. T. (2016). Identification and characterization of parasitism genes from the pinewood nematode *Bursaphelenchus xylophilus* reveals a multilayered detoxification strategy. *Mol. Plant Pathol.* 17 286–295. 10.1111/mpp.12280 25981957 PMC6638532

[B14] Filipiak A. Malewski T. Matczyńska E. Tomalak M. (2020). Molecular variation among virulent and avirulent strains of the quarantine nematode *Bursaphelenchus xylophilus*. *Mol. Genet. Genomics.* 10.1007/s00438-020-01739-w 33169231 PMC7895788

[B15] Futai K. (2013). Pine wood nematode, *Bursaphelenchus xylophilus*. *Annu. Rev. Phytopathol.* 51 61–83. 10.1146/annurev-phyto-081211-172910 23663004

[B16] Geldhof P. Visser A. Clark D. Saunders G. Britton C. Gilleard J. (2007). RNA interference in parasitic helminths: current situation, potential pitfalls and future prospects. *Parasitology* 134 609–619. 10.1017/S0031182006002071 17201997

[B17] Haegeman A. Mantelin S. Jones J. T. Gheysen G. (2012). Functional roles of effectors of plant-parasitic nematodes. *Gene* 492 19–31. 10.1016/j.gene.2011.10.040 22062000

[B18] Hammond S. M. Boettcher S. Caudy A. A. Kobayashi R. Hannon G. J. (2001). Argonaute2, a link between genetic and biochemical analyses of RNAi. *Science* 293 1146–1150. 10.1126/science.1064023 11498593

[B19] Han Z. M. Hong Y. D. Zhao B.G. (2003). A study on pathogenicity of bacteria carried by pine wood nematodes. *J. Phytopathol.* 151 683–689. 10.1046/j.1439-0434.2003.00790.x

[B20] Hannun Y. A. Obeid L. M. (2008). Principles of bioactive lipid signalling: lessons from sphingolipids. *Nat. Rev. Mol. Cell Biol.* 9 139–150. 10.1038/nrm2329 18216770

[B21] Hu L. J. Wu X. Q. Li H. Y. Zhao Q. Wang Y. C. Ye J. R. (2019). An effector, BxSapB1, induces cell death and contributes to virulence in the pine wood nematode *Bursaphelenchus xylophilus*. *Mol. Plant Microbe Interact.* 32 452–463. 10.1094/MPMI-10-18-0275-R 30351223

[B22] Ichihara Y. Fukuda K. Suzuki K. (2000). Early symptom development and histological changes associated with migration of *Bursaphelenchus xylophilus* in seedling tissues of *Pinus thunbergii*. *Plant Dis.* 84 675–680. 10.1094/PDIS.2000.84.6.675 30841110

[B23] Ikeda T. (1996). Responses of water-stressed *Pinus thunbergii* to inoculation with avirulent pine wood nematode (*Bursaphelenchus xylophilus*): water relations and xylem histology. *J. For. Res.* 1 223–226. 10.1007/BF02348329

[B24] Kang J. S. Koh Y. H. Moon Y. S. Lee S. H. (2012). Molecular properties of a venom allergen-like protein suggest a parasitic function in the pinewood nematode *Bursaphelenchus xylophilus*. *Int. J. Parasitol.* 42 63–70. 10.1016/j.ijpara.2011.10.006 22142561

[B25] Kawazu K. Zhang H. Yamashita H. Kanzaki H. (1996). Relationship between the pathogenicity of the pinewood nematode, *Bursaphelenchus xylophilus*, and phenylacetic acid production. *Biosci. Biotechnol. Biochem.* 60 1413–1415. 10.1271/bbb.60.1413 8987588

[B26] Kettles G. J. Bayon C. Canning G. Rudd J. J. Kanyuka K. (2017). Apoplastic recognition of multiple candidate effectors from the wheat pathogen *Zymoseptoria tritici* in the nonhost plant *Nicotiana benthamiana*. *New Phytol.* 213 338–350. 10.1111/nph.14215 27696417 PMC5132004

[B27] Kikuchi T. Cotton J. A. Dalzell J. J. Hasegawa K. Kanzaki N. McVeigh P. (2011). Genomic insights into the origin of parasitism in the emerging plant pathogen *Bursaphelenchus xylophilus*. *PLoS Pathog.* 7:e1002219. 10.1371/journal.ppat.1002219 21909270 PMC3164644

[B28] Kikuchi T. Jones J. T. Aikawa T. Kosaka H. Ogura N. (2004). A family of glycosyl hydrolase family 45 cellulases from the pine wood nematode *Bursaphelenchus xylophilus*. *FEBS Lett.* 572 201–205. 10.1016/j.febslet.2004.07.039 15304348

[B29] Kikuchi T. Shibuya H. Aikawa T. Jones J. T. (2006). Cloning and characterization of pectate lyases expressed in the esophageal gland of the pine wood nematode *Bursaphelenchus xylophilus*. *Mol. Plant Microbe Interact.* 19 280–287. 10.1094/MPMI-19-0280 16570658

[B30] Kirino H. Yoshimoto K. Shinya R. (2020). Thaumatin-like proteins and a cysteine protease inhibitor secreted by the pine wood nematode *Bursaphelenchus xylophilus* induce cell death in *Nicotiana benthamiana*. *PLoS One* 15:e0241613. 10.1371/journal.pone.0241613 33125444 PMC7598465

[B31] Kiyohara T. (1989). Etiological study of pine wilt disease. *Bull. For. For. Prod. Res. Instit.* 353 127–176.

[B32] Kiyohara T. Bolla R. I. (1990). Pathogenic variability among populations of the pinewood nematode, *Bursaphelenchus Xylophilus*. *For. Sci.* 36 1061–1076. 10.1093/forestscience/36.4.1061

[B33] Kiyohara T. Tokushige Y. (1971). Inoculation experiments of a nematode, *Bursaphelenchus* sp., onto pine trees. *J. Jpn. For. Soc.* 53 210–218. 10.11519/jjfs1953.53.7_210

[B34] Knox D. P. Geldhof P. Visser A. Britton C. (2007). RNA interference in parasitic nematodes of animals: a reality check? *Trends Parasitol.* 23 105–107. 10.1016/j.pt.2007.01.007 17276139

[B35] Ma H. B. Lu Q. Liang J. Zhang X. (2011). Functional analysis of the cellulose gene of the pine wood nematode, *Bursaphelenchus xylophilus*, using RNA interference. *Genet. Mol. Res.? GMR* 10 1931–1941. 10.4238/vol10-3gmr1367 21948755

[B36] Morisaka H. Kirino A. Kobayashi K. Ueda M. (2012). Two-Dimensional protein separation by the HPLC system with a monolithic column. *Biosci. Biotech. Biochem.* 76 585–588. 10.1271/bbb.110770 22451405

[B37] Mota M. M. Braasch H. Bravo M. A. Penas A. C. Burgermeister W. Metge K. (1999). First report of *Bursaphelenchus xylophilus* in Portugal and in Europe. *Nematology* 1 727–734. 10.1163/156854199508757

[B38] Nelson B. K. Cai X. Nebenführ A. (2007). A multicolored set of *in vivo* organelle markers for co-localization studies in *Arabidopsis* and other plants. *Plant J.* 51 1126–1136. 10.1111/j.1365-313X.2007.03212.x 17666025

[B39] Odani K. Sasaki S. Yamamoto N. Nishiyama Y. Tamura H. (1985). Differences in dispersal and multiplication of two associated nematodes, *Bursaphelenchus xylophilus* and *Bursaphelenchus mucronatus* in pine seedlings in relation to the pine wilt disease development. *J. Jpn. For. Soc.* 67 398–403. 10.11519/jjfs1953.67.10_398

[B40] Ong S.-E. Blagoev B. Kratchmarova I. Kristensen D. B. Steen H. Pandey A. (2002). Stable isotope labeling by amino acids in cell culture, SILAC, as a simple and accurate approach to expression proteomics. *Mol. Cell. Proteomics* 1 376–386. 10.1074/mcp.M200025-MCP200 12118079

[B41] Palomares-Rius J. E. Tsai I. J. Karim N. Akiba M. Kato T. Maruyama H. (2015). Genome-wide variation in the pinewood nematode *Bursaphelenchus xylophilus* and its relationship with pathogenic traits. *BMC Genomics* 16:845. 10.1186/s12864-015-2085-0 26493074 PMC4619224

[B42] Ross P. L. Huang Y. N. Marchese J. N. Williamson B. Parker K. Hattan S. (2004). Multiplexed protein quantitation in *Saccharomyces cerevisiae* using amine-reactive isobaric tagging reagents. *Mol. Cell. Proteomics MCP* 3 1154–1169. 10.1074/mcp.M400129-MCP200 15385600

[B43] Santos C. S. Pinheiro M. Silva A. I. Egas C. Vasconcelos M. W. (2012). Searching for resistance genes to *Bursaphelenchus xylophilus* using high throughput screening. *BMC Genomics* 13:599. 10.1186/1471-2164-13-599 23134679 PMC3542250

[B44] Saucedo-García M. Guevara-García A. González-Solís A. Cruz-García F. Vázquez-Santana S. Markham J. E. (2011). MPK6, sphinganine and the LCB2a gene from serine palmitoyltransferase are required in the signaling pathway that mediates cell death induced by long chain bases in *Arabidopsis*. *New Phytol.* 191 943–957. 10.1111/j.1469-8137.2011.03727.x 21534970

[B45] Shaffer J. P. (1995). Multiple Hypothesis Testing. *Annu. Rev. Psychol.* 46 561–584.

[B46] Shinya R. Takeuchi Y. Futai K. (2009). A technique for separating the developmental stages of the propagative form of the pine wood nematode (*Bursaphelenchus xylophilus*). *Nematology* 11 305–307. 10.1163/156854108X399164

[B47] Shinya R. Morisaka H. Takeuchi Y. Ueda M. Futai K. (2010). Comparison of the surface coat proteins of the pine wood nematode appeared during host pine infection and *in vitro* culture by a proteomic approach. *Phytopathol.* 100 1289–1297. 10.1094/PHYTO-04-10-0109 21062170

[B48] Shinya R. Takeuchi Y. Ichimura K. Takemoto S. Futai K. (2012). Establishment of a set of inbred strains of the pine wood nematode, *Bursaphelenchus xylophilus* (Aphelenchida: Aphelenchoididae), and evidence of their varying levels of virulence. *Appl. Entomol. Zool.* 47 341–350. 10.1007/s13355-012-0124-8

[B49] Shinya R. Morisaka H. Kikuchi T. Takeuchi Y. Ueda M. Futai K. (2013). Secretome analysis of the pine wood nematode *Bursaphelenchus xylophilus* reveals the tangled roots of parasitism and its potential for molecular mimicry. *PLoS One* 8:e67377. 10.1371/journal.pone.0067377 23805310 PMC3689755

[B50] St John F. J. González J. M. Pozharski E. (2010). Consolidation of glycosyl hydrolase family 30: a dual domain 4/7 hydrolase family consisting of two structurally distinct groups. *FEBS Lett.* 584 4435–4441. 10.1016/j.febslet.2010.09.051 20932833

[B51] Vicente C. S. L. Ikuyo Y. Shinya R. Mota M. Hasegawa K. (2015). Catalases induction in high virulence pinewood nematode *Bursaphelenchus xylophilus* under hydrogen peroxide-induced stress. *PLoS One* 10:e0123839. 10.1371/journal.pone.0123839 25894519 PMC4404050

[B52] Viney M. E. Thompson F. J. (2008). Two hypotheses to explain why RNA interference does not work in animal parasitic nematodes. *Int. J. Parasitol.* 38 43–47. 10.1016/j.ijpara.2007.08.006 18028931

[B53] Yi C. K. Byun B. H. Park J. D. Yang S. I. Chang K. H. (1989). First finding of the pine wood nematode, *Bursaphelenchus xylophilus* (Steiner et Buhrer) Nickle and its insect vector in Korea. *Res. Rep. For. Res. Inst.* 38 141–149.

[B54] Zhao B. G. Lin F. (2005). Mutualistic symbiosis between *Bursaphelenchus xylophilus* and bacteria of the genus *Pseudomonas*. *For. Pathol.* 35 339–345. 10.1111/j.1439-0329.2005.00417.x

[B55] Zhou Z. Sakaue D. Wu B. Hogetsu T. (2007). Genetic structure of populations of the pinewood nematode *Bursaphelenchus xylophilus*, the pathogen of pine wilt disease, between and within pine forests. *Phytopathology* 97 304–310. 10.1094/PHYTO-97-3-0304 18943649

