# Supplementary figures and images for: Comparative Secretome and Functional Analyses Reveal Glycoside Hydrolase Family 30 and Cysteine Peptidase as Virulence Determinants in the Pinewood Nematode Bursaphelenchus xylophilus
Source: Front Plant Sci. 2021 Mar 8;12:640459. doi: 10.3389/fpls.2021.640459 (PMC7982738; doi:10.3389/fpls.2021.640459)

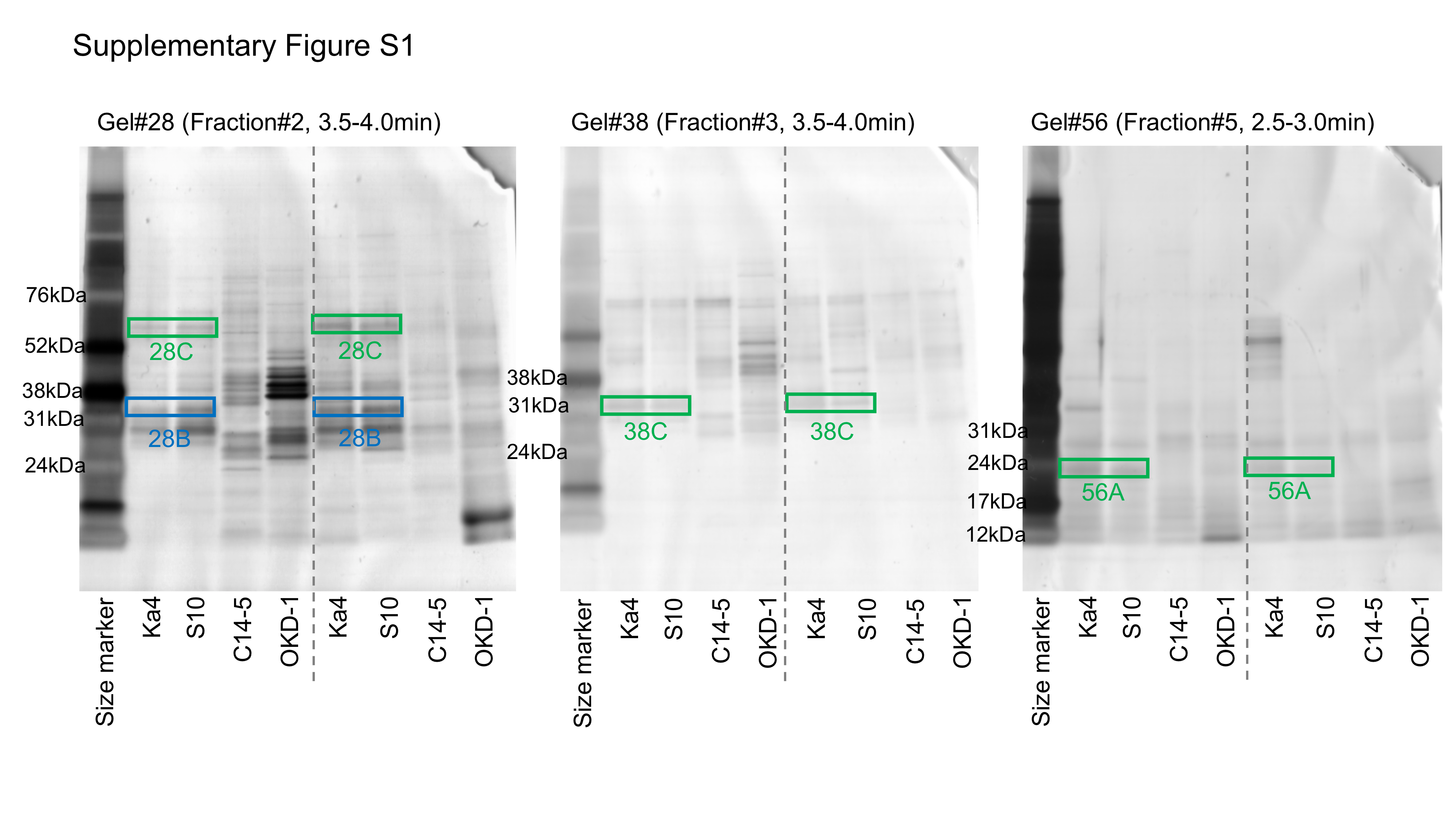

Supplement: Supplementary Figure 2 — SDS-PAGE comparative analysis of B. xylophilus proteins derived from 2D-HPLC fractions. Each fraction was run in a sequential manner for the four isolates of B. xylophilus (Ka4, S10, C14-5, and OKD-1). [file Image_1.TIFF]

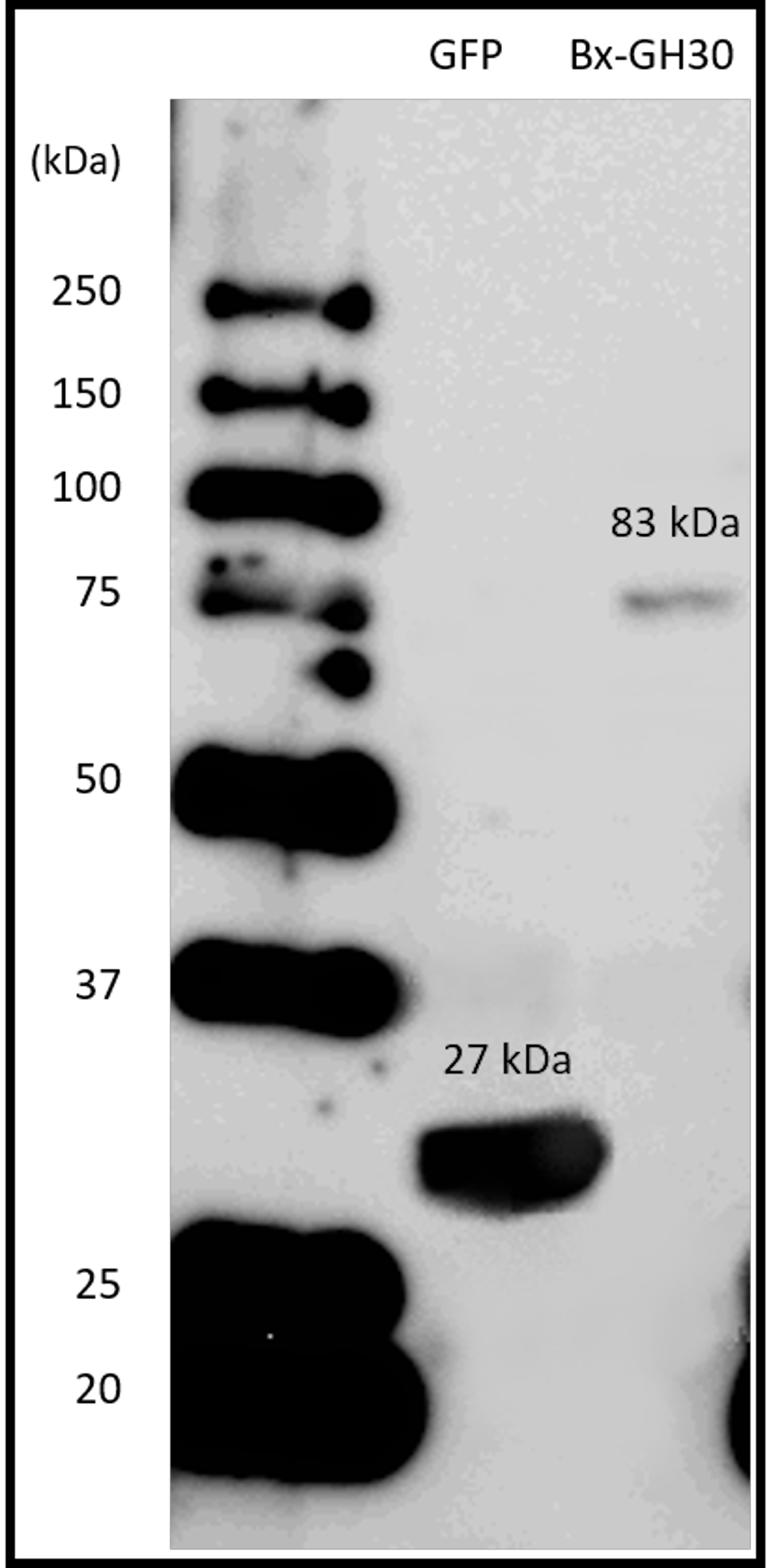

Supplement: Supplementary Figure 2 — Bands of Bx-GH30 on Western blot membranes. GFP-only vector control protein and Bx-GH-30-GFP fusion protein expressed in N. benthamiana were detected with anti-GFP antibodies. The GFP control band is approximately 27 kDa. Bx-GH30 band is approximately 83 kDa. [file Image_2.TIF]

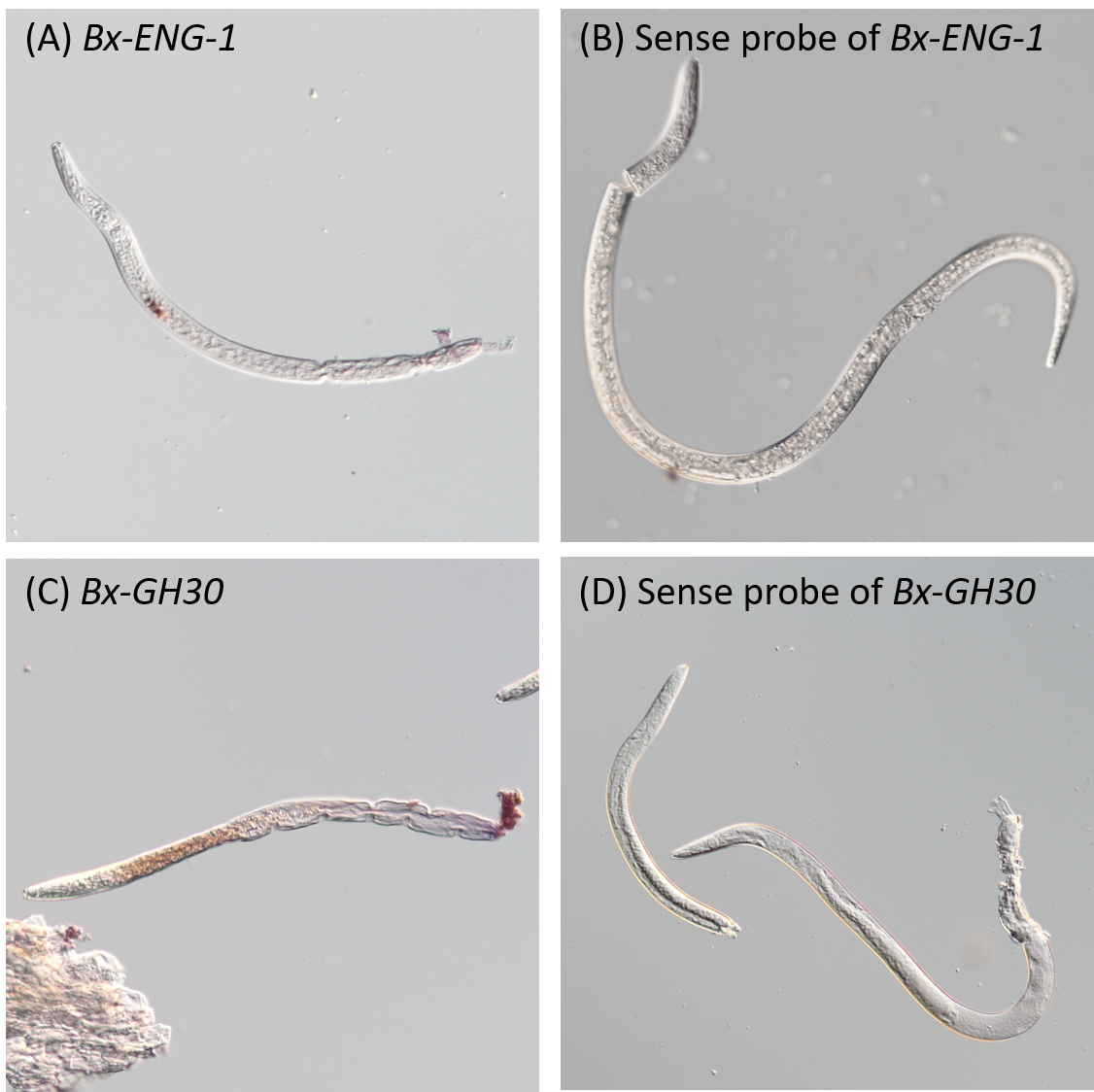

Supplement: Supplementary Figure 3 — mRNA localization of Bx-GH30 in nematodes. The localization of Bx-ENG-1 (A) as a positive control for gene expression in pharyngeal gland cells, after in situ hybridization. Localization of transcripts encoding Bx-GH30 of the pine wood nematode around pharyngeal glands, after in situ hybridization (C). No signals were produced using the sense probes for Bx-ENG-1 (B) and Bx-GH30 (D). [file Image_3.TIF]

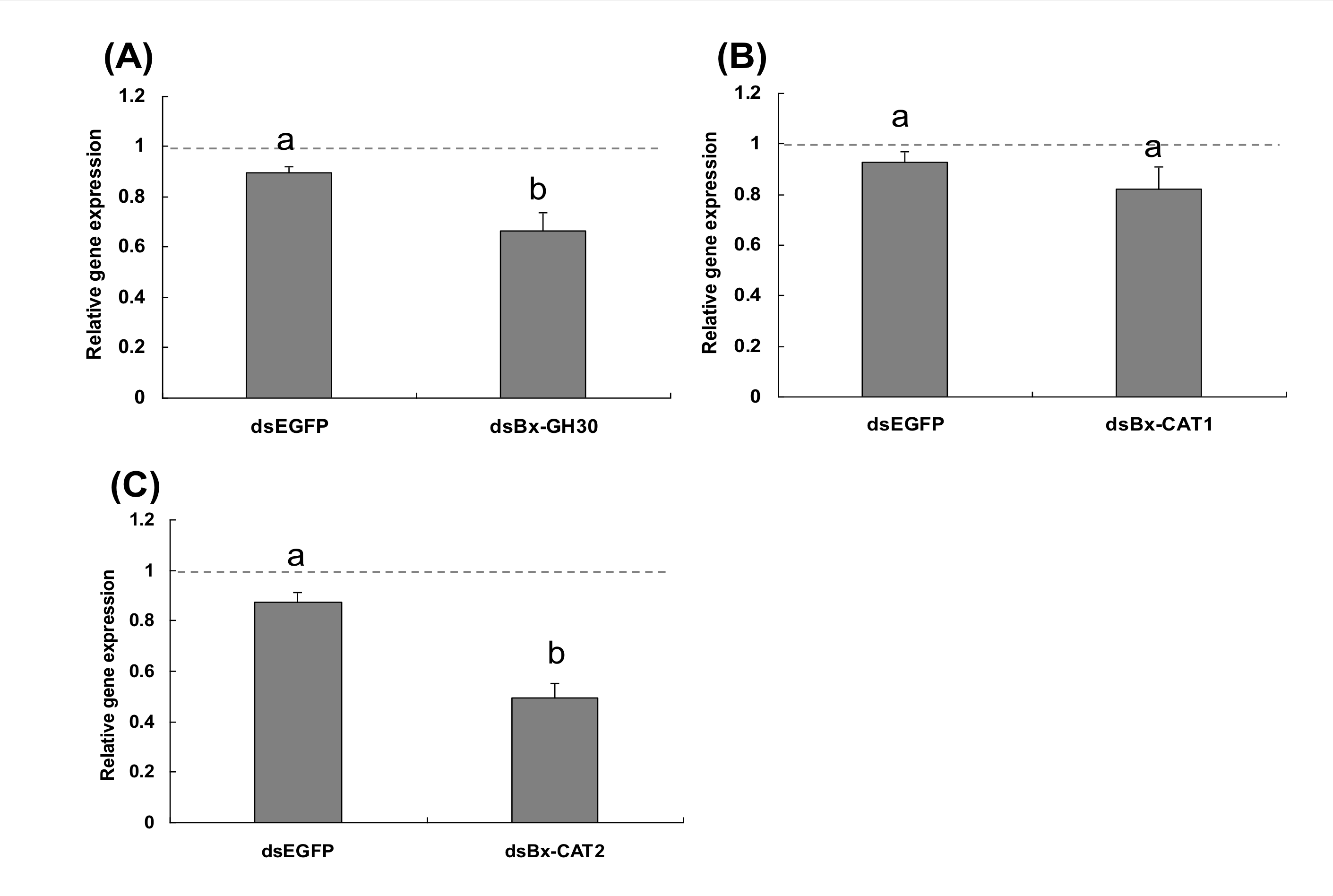

Supplement: Supplementary Figure 4 — mRNA expression levels of Bx-GH30 (A), Bx-CAT1 (B), and Bx-CAT2 (C) genes in B. xylophilus after soaking in target dsRNA solution and EGFP dsRNA solution (a non-endogenous gene), relative to the control level (non-dsRNA treatment). Each bar represents the mean ± SD of three biological replicates. The expression level in the control was regarded as 1.0. Different letters indicate significant differences between genes (P < 0.01, Student’s t-test). [file Image_4.TIFF]
